# How are inequalities generated in the management and consequences of gastrointestinal infections in the UK? An ethnographic study

**DOI:** 10.1016/j.socscimed.2021.114131

**Published:** 2021-08

**Authors:** Suzanne Rotheram, Jessie Cooper, Ben Barr, Margaret Whitehead

**Affiliations:** a National Institute of Health Research Health Protection Research Unit in Gastrointestinal Infections, The University of Liverpool, Waterhouse Building (2nd Floor, Block F), 1-5 Brownlow Street, Liverpool, L69 3GL, United Kingdom; b Department of Public Health, Policy and Systems, The University of Liverpool, Whelan Building, Liverpool, L68 3GB, United Kingdom; c Division of Health Services Research and Management, School of Health Sciences, City, University of London, Myddelton Street Building, London, EC1R 1UW, United Kingdom

**Keywords:** Gastrointestinal infection, Health inequalities, Behavioural interventions, Ethnography, COVID-19

## Abstract

Gastrointestinal infections are an important global public health issue. In the UK, one in four people experience a gastrointestinal infection each year and epidemiological research highlights inequalities in the burden of disease. Specifically, poorer children are at greater risk of infection and the consequences of illness, such as symptom severity and time off work/school, are greater for less privileged groups of all ages. Gastrointestinal infections are, however, largely ‘hidden’ within the home and little is known about the lived experience and practices surrounding these illnesses, how they vary across contrasting socioeconomic contexts, or how inequalities in the disease burden across socioeconomic groups might come about. This paper presents data from an ethnographic study which illuminate how socioeconomic inequalities in the physical and material management and consequences of gastrointestinal infections are generated in families with young children. The study shows how the ‘work’ needed to manage gastrointestinal infections is more laborious for people living in more ‘disadvantaged’ conditions, exacerbated by: more overcrowded homes with fewer washing and toilet facilities; inflexible employment; low household incomes; and higher likelihood of co-morbidities which can be made worse by having a gastrointestinal infection. Our findings call into question the current approach to prevention of gastrointestinal infections which tend to focus almost exclusively on individual behaviours, which are not adapted to reflect differences in socioeconomic context. Public health agencies should also consider how wider social, economic and policy contexts shape inequalities in the management and consequences of illness. Our findings are also pertinent to the COVID-19 pandemic response in the UK. They highlight how research and policy approaches to acute infectious diseases need to take into consideration the differing lived experiences of contrasting households if they wish to address (and avoid exacerbating) inequalities in the future.

## Highlights

• First ethnographic study to examine UK socioeconomic inequalities in GI infections.
• GI infections create a huge amount of labour for all families with young children.
• Co-morbidities, poorer living conditions and low income increase GI infection labour.
• Low paid, insecure, inflexible work exacerbates material consequences of infection.
• GI infection policy should look beyond behavioural advice to address inequalities.

## 1 Introduction

Gastrointestinal (GI) infections are a group of acute infectious diseases of the gastrointestinal tract caused by viruses, bacteria or parasites transmitted through contaminated food or water, the environment, contact with animals, or contact with another person who is ill (FSA, 2012). Symptoms of having a GI infection include vomiting, diarrhoea, fever and abdominal pain (FSA, 2012) and while symptoms often resolve without complications, they can progress to more serious consequences such as dehydration and death, particularly in the very elderly, very young, or those with pre-existing health conditions (Lund and O'Brien, 2011). In high income countries such as the UK, where the vast majority of the population have access to healthcare, sanitation and clean water, mortality due to GI infections is low but morbidity is high, with one quarter of the UK population experiencing at least one GI infection each year (FSA, 2012). The financial cost to individuals in terms of medication and time off work, to the National Health Service (NHS) and to the economy as a consequence of this common disease make GI infections an important public health issue (FSA, 2012). However, as most people recover without medical intervention, many GI infections are characterised medically as a ‘mild’, ‘self-limiting’ illness (Tam et al., 2012).

To prevent GI infections in the home, community and health services UK public health advice and policy largely focus on individual behavioural practices. For example, to prevent foodborne illness, public health messages promote specific kitchen hygiene practices (FSA, 2020). To reduce GI infections spread in the community, public health policies state that adults and children should stay away from work and school until at least 48 hours after symptoms of vomiting and diarrhoea finish and advise increasing cleaning and hand washing in the home (NHS, 2020a). To prevent infections spreading within formal health services, the NHS advises people to manage illness at home and only seek medical advice for very young children, if parents are worried about dehydration in their child or if symptoms deteriorate or last a long time (NHS, 2020a). One of the consequences of these messages is that the majority of GI infections are not reported to UK public health surveillance (FSA, 2012). GI infections are often, therefore, ‘hidden’ within the home: GI surveillance data under-estimates the actual number of GI infections in the community (FSA, 2012) and little is known about the lived experience of illness or the practices used to manage this common infectious disease. A recent systematic review showed that the majority of UK qualitative research engaging with GI infections has aligned with the dominant individualistic public health approach by asking how behaviours might shape risk, rather than examining the lived experience or consequences of GI infections (Rotheram et al., 2020).

The paucity of qualitative research exploring the lived experience of GI infections is particularly concerning in light of increasing evidence for socioeconomic inequalities in relation to GI infections (Adams et al., 2018). Epidemiological studies from the UK show that the risk of having a GI infection is greater for children from what are defined as ‘disadvantaged’ areas compared to their more ‘advantaged’ counterparts, particularly with respect to hospital admission (Olowokure et al., 1999; Pockett et al., 2011). There are also inequalities in the consequences of having a GI infection such as symptom severity and time taken off work/school, with more severe symptoms and higher rates of absence reported in less privileged groups of all ages (Rose et al., 2017). Until recently inequalities like these, in the causes and consequences of common infectious diseases, have been under-recognised in the UK. This has been thrown into stark relief with the COVID-19 pandemic following a similar pattern as seen for GI infections and disproportionately affecting areas defined as ‘disadvantaged’ (ONS, 2020). This paper therefore aims to make visible the lived experience and practices surrounding GI infections and examine how inequalities in the management and consequences of illness are shaped within different socioeconomic settings. To do this, we utilise data from an ethnographic study which focused on the experience of families with young children managing GI infections in their homes in two socioeconomically contrasting places. In this paper, the qualitative data reveal how misleading the common description of GI infections as a ‘mild’, ‘self-limiting’ illness is, and makes visible the considerable extent and nature of the work needed to manage illness in the home. The data also show how inequalities in this labour are unequally shaped by contrasting socioeconomic contexts. We use the term ‘work’ to refer to the different kinds of labour which get used within the everyday management of illness (Corbin and Strauss, 1985). Before presenting the analysis of these data, we outline social science critiques of the use of behavioural approaches to address health inequalities and the relative neglect of lay knowledge. We justify our use of an ethnographic approach to understand the lived experiences and practices surrounding GI infections and how inequalities are shaped by contrasting social, economic and environmental contexts. These critiques and methodology underpin our study and enable a novel focus for us to approach inequalities in GI infections in the UK context.

## 2 How behavioural approaches render invisible the ways in which socio-economic conditions shape differences in health

As yet there is an absence of critiques of behavioural interventions with respect to GI infections in the UK. However, social science critiques of individual behavioural approaches and their failure to address socioeconomic inequalities in relation to non-communicable diseases (NCDs) are well established, including in relation to cardiovascular disease, cancer, diabetes and chronic respiratory disorders (e.g. Baum and Fisher, 2014; Whitehead and Popay, 2010; Williams and Fullagar, 2019). Commentators argue that interventions designed to change individual behaviours have had limited success in reducing inequalities because they do not consider how wider structural factors, such as people's living and working conditions, can constrain individual ‘choices’ around these behaviours (Alvaro et al., 2011; Baum and Fisher, 2014). Focusing solely on individual behaviours can therefore render invisible the ways in which socio-economic inequalities shape differences in health.

A related critique points to a common, if flawed, assumption underlying traditional behavioural approaches: that the main barrier to behaviour change is lack of knowledge or skills and providing this missing information will motivate individuals to change their behaviour (Baum and Fisher, 2014). Too few studies test such assumptions by learning from lay people about their day-to-day experiences managing health. Popay and others call for the evidential value of lay knowledge to be recognised, especially with respect to tackling inequalities in health (Popay et al., 1998).

A further important critique highlights how these same interventions ‘moralise’ particular health conditions, thereby shifting the responsibility to follow this advice on to individuals, and ultimately ‘blaming’ them for their own ill health (Bell et al., 2009). Bell and colleagues highlight this moral dimension using the example of how mothers (often working class, single or minority ethnic mothers) have been made to feel solely responsible for their children's exposure to smoking, excessive eating and alcohol while in utero (Bell et al., 2009). Social scientists have therefore called for a more ‘upstream’ approach to addressing inequalities, i.e. one that focuses on the political, social and economic causes of inequalities in disease rather than solely on individual behaviours (Dahlgren and Whitehead, 2007).

These behavioural approaches to NCDs have many things in common with the official focus of interventions to reduce rates of GI infections in the UK, which concentrate on promoting individual behaviours, such as hygiene and isolation. Individual behavioural approaches like these have so far proved ineffective at reducing socioeconomic inequalities in GI infections, which may even be widening (Adams et al., 2018; Rose et al., 2017). These individualistic policy approaches may also, in the process, inadvertently stigmatise those they intend to benefit. There is already some evidence of individuals being ‘blamed’ by professionals for acquiring foodborne GI infections through poor kitchen hygiene practices (Meah, 2014).

A similar critique to that posed by social science research into NCDs has been raised to the response to the COVID-19 pandemic, whereby control measures have targeted individual behaviours such as washing hands and social isolation (NHS, 2020b) and have overlooked the structural factors such as poverty which lead to inequalities in the risk and consequences of COVID-19 (Whitehead et al., 2021). Research that understands the wider, situated context of illness, recognises the value of lay knowledge and avoids ‘blaming’ communities is vital to better understand inequalities in GI infections and other infectious diseases such as COVID-19.

To help address this gap this paper takes an ethnographic approach to better understand how the relationship between inequality and disease can be situated within its wider social, economic, cultural and political context (Kierans et al., 2013). Ethnographic approaches have been effectively used in a UK context to examine health inequalities during government austerity policies (Garthwaite and Bambra, 2018) and are transferrable to our interest in how wider contexts might shape socio-economic differences in GI infections. Below, we outline the ethnographic methodology for this study, before reporting the findings. In the discussion we reflect on how its insights may be relevant not only to GI infections but also to COVID-19.

## 3 Methodology

This paper draws on data from a wider study which aimed to examine how the management and consequences of UK gastrointestinal infections are shaped in the context of households with young children in socioeconomically contrasting places. In this paper, we tackle the research question of how the observed socioeconomic inequalities in the consequences of GI infections in children might come about. To do this, we compare and contrast ethnographic data from families with young children living in two socioeconomically contrasting areas in the North West of England. These areas were chosen using locally available health and demographic statistics, as well as insights from Environmental Health Officers working in the local authority and criteria from previous comparative studies (e.g. Maciver and Macintyre, 1987; Popay et al., 2003). These considerations led us to choose two socioeconomically contrasting urban areas, the size of wards, from the same local authority. The two areas had similar population sizes and ethnic mix (95% white British), but differed in their socioeconomic conditions and health profiles (contrasts described in the results). We purposely chose areas that were not adjacent, so that they did not share services, in case differences in access to services were pertinent to our question on how inequalities were generated. The relatively ‘disadvantaged’ area we call ‘Rockport’ and the relatively ‘advantaged’ place we call ‘Seaview’. The terms ‘advantaged’ and ‘disadvantaged’ are placed in inverted commas throughout this paper to reflect the understanding that, while these categorisations are terms commonly used within the literature and have been made necessary to discuss inequalities, they are also socially constructed (Bowker and Star, 2000). The judgement that these terms bring with them may not, therefore, be recognised by the people living in the places to which they refer (Popay et al., 2003).

The study was given ethical approval by the University of Liverpool in February 2017 (Reference 0915) and our ethical reflections can be found in the discussion. Data were collected by SR over 10 months from March–December 2017 and included participant and non-participant observations (around 150 h), ethnographic interviews (n = 13), and narrative interviews (n = 23). Ethnographic fieldwork concentrated on participating in the everyday activities and social lives of people living in the research areas (Singer, 2016) to gain insights into the lived experiences of GI infections and the practices used to manage illness in the socio-economically contrasting study areas. Observations and ethnographic interviews took place while: volunteering at playgroups and community groups for families with young children; attending a local history group; spending time with families of young children who had experienced a recent GI infection and ‘hanging out’ (Hammersley and Atkinson, 2007) in community centres. Ethnographic interviews captured spontaneous conversations during fieldwork which were relevant to our research question and complemented formal interview and observation data. Formal interviews took a narrative format to understand the lived experience of GI infections and practices surrounding illness (Riessman, 2008). Interviews were conducted with parents of pre-school children (under the age of five) living in the study areas, who had experienced a GI infection (defined using a symptom-based definition (Tam et al., 2012)) in the previous 12 months. Four male and nineteen female participants took part in narrative interviews. Sampling was purposive and aimed to recruit men and women from households with different interactions with healthcare - from those whose children had been admitted to hospital to households with no healthcare contact (Table 1). The recruitment of more women than men reflected the higher proportion of women in the locations where recruitment took place (nurseries and community groups). Interviews were audio recorded and transcribed. Participants who participated in formal interviews or who worked in groups where participant observations were conducted were provided with information sheets and gave written, informed consent to taking part. Participants who gave ethnographic interviews provided verbal consent to being part of the study. Casual conversations in the community were included as non-verbatim data in field notes. Pseudonyms were used for participants and places to protect the identity of participants.

**Table 1:** Interview participants’ details for Seaview and Rockport.

Pseudonym of participant	Area^a^	Male/Female	Age of child(ren)	Employment	Engagement with Healthcare^b^
Zoe	Rockport	F	1	p/t Care assistant	None
Jo	Rockport	F	2 & 6	Full-time mum	Doctor
Naomi	Rockport	F	2	p/t Cleaner	Emergency Department
Lydia	Rockport	F	1 & 2	Full-time mum	None
Georgia	Rockport	F	2	Full-time mum	Doctor
Talia^c^	Rockport	F	2	Full-time mum	NHS 111
Scott^c^		M		Disability benefit	
Mia	Rockport	F	2	Care-assistant	Doctor & Chemist
Freya	Rockport	F	3	Full-time mum	Emergency Department
Caren	Rockport	F	1 & 2	p/t shop assistant	Doctor
Sarah	Seaview	F	2 & 5	p/t Manager	None
Linzi	Seaview	F	1 & 1	Full-time mum	None
Holly	Seaview	F	2	p/t civil servant	Doctor
Esme^c^	Seaview	F	1	p/t Academic	None
Neil^c^		M		Academic	
Annabelle	Seaview	F	2 & 4	p/t Call centre	None
Penny	Seaview	F	<1, 2 & 4	p/t Doctor	None
Stephanie^c^	Seaview	F	<1, 2 & 4	Full-time mum	Online advice
Ed^c^		M		Engineer	
Harriett	Seaview	F	1, 4 & 7	Full-time mum	None
Jess	Seaview	F	3	p/t self-employed	Online advice
Jack^c^	Seaview	M	<1 & 3	Doctor	None
Lucy^c^		F		Doctor	

NHS 111 is an NHS telephone advice service.

a Rockport = relatively ‘disadvantaged’, Seaview = relatively ‘advantaged’.

b Engagement with health care for child's most recent episode of a GI infection.

c Denotes two parents co-parenting a child.

These multiple methods allowed for an understanding of the relationship between inequality and disease in three ways. Firstly, they gave access to understanding what people do, how they understand these actions and how these actions fit into their economic, cultural, social and political contexts. Secondly, they allowed for a consideration of how broader political and socioeconomic structures might play a role in how well GI infections could be managed in the home. Lastly it took into account connections between different arenas of social life (e.g. work, illness and family) rather than studying them separately (Kierans et al., 2013).

Data analysis took place throughout the research process (Hammersley and Atkinson, 2007). Once fieldwork had finished a more formal thematic analysis was conducted on the data (Braun and Clarke, 2006), which is a well-recognised and documented type of analysis for narrative interviews (Riessman, 2004), and facilitated our analysis across the different types of data. Data were read through and ‘codes’ or, ‘units of meaning’ were assigned to a sentence or paragraph of the data according to how they captured particular meanings or concepts (Miles and Huberman, 1994). NVIVO 11 was used to organise the data and coding was completed for the whole dataset by SR. Iterative themes, linked to the data, were then developed from these codes by discussion between SR, MW, JC and BB (Rapley in Silverman, 2011). During analysis we paid particular attention to differences between Seaview and Rockport so that contrasts between the experiences and practices of residents living in the two areas could be drawn out (Popay et al., 2003).

In the following results, we first provide context to the setting of the research: Rockport and Seaview. We then show the huge amount of ‘hidden’ work expended to manage GI infections and demonstrate inequalities in this labour. Finally, we show how following behavioural advice around social isolation and exclusion (from places of work, nurseries and schools) creates inequalities in the financial consequences of this illness.

## 4 Results

### 4.1 Contrasting places: introducing ‘Rockport’ and ‘Seaview’

Rockport and Seaview both grew from fishing ports but have developed very differently over the last 50 years. Rockport has lost extensive local industry and jobs, whereas, in contrast, Seaview has attracted professionals (such as teachers, doctors and academics) who have chosen to live in Seaview and commute to work in local towns and cities. Local statistics gave an indication of the impact that these contrasting trajectories of development have had. In 2017 Seaview's life expectancy, was, on average, five years longer than Rockport. Over 95% of Rockport's population compared with none of Seaview's population were classified as living in in the most deprived 20% of areas in the UK. Approximately 47% of children in Rockport were living in poverty (defined as household income less than 60% of current median income) compared to 7% of children living in Seaview. The percentage of 16–64 year olds living with long-term medical conditions in Rockport was more than double that of Seaview (ONS, 2016) and the pooled 3-year rate of hospitalisations per 100,000 population as a consequence of having a GI infection in Rockport was almost twice that of Seaview. The relative ‘disadvantage’ of Rockport and the people who lived there compared to Seaview and its residents was therefore evident in multiple areas of life, from life expectancy through to area-level deprivation, income and health.

Fieldwork, conducted in 2017, took place after seven years of UK government austerity policies and the rollout of a new welfare payment, ‘Universal credit’ (gov.uk, 2019a). Both these policies - austerity which has reduced public expenditure by cutting local government budgets, and Universal Credit, which has amalgamated welfare payments for people on a low income or out of work - have had a disproportionately negative impact on post-industrial communities like Rockport with high levels of unemployment, welfare and poverty (e.g. Cheetham et al., 2019; Reed and Portes, 2018). Stark differences in terms of the income of local residents and local authority investment between the areas were evident throughout fieldwork and are described in the observations below.

In Rockport, many shops on the high-street were boarded up in the middle of the day, playgrounds were poorly maintained and a local government-funded centre for families had been closed. Housing in Rockport was largely made up of estates with small terraced, or semi-detached houses built close to each other, typical of the kind of houses found in a council estate built in the 1950s. Many residents in Rockport lived on low incomes which did not allow for household repairs: one woman described how she had been washing her clothes by hand since her washing machine broke six months ago; another resident's home had a large hole through her back door and wall-paper peeled off the walls to waist level. Community workers in the area described how the rollout of Universal Credit had led to an increase in the use of foodbanks (local charity provision of groceries for households unable to afford to buy food). The lack of government investment and the historical decline in industry had led to community groups stepping in to plug gaps between family income and the cost of living. Rockport's community centres provided access to free or low-cost food including: a food store where groceries could be exchanged for a donation; meals for residents who couldn't afford to pay for the electricity or gas needed to cook; a reasonably priced café, social supermarket; and a packed-lunch and free breakfast provided for local children whose families struggled to afford food in the holidays.

In contrast, homes in Seaview were often large, with multiple bedrooms and bathrooms. Many houses had wide driveways, manicured hedges and multiple cars (sometimes a horsebox, caravan or boat) parked outside. Its bustling high street had multiple independent cafes, restaurants and shops selling organic food, designer clothes and antiques in amongst the usual UK charity shops, takeaways, betting shops and supermarkets. The ward was served by numerous well-maintained and well-equipped children's playgrounds and parks bordered by benches and newly planted flowers. Seaview's local community centre ran a café and rented out its rooms to people who wished to organise and run events. Unlike Rockport, Seaview's community centre did not run a social supermarket, food store, or provide free meals for children in the holidays, suggesting that the income of residents generally allowed them to afford the cost of living without community groups stepping in to help.

These insights from fieldwork show the stark contrasts between two places within the same local authority and the circumstances under which their residents lived. These contrasting social, economic and place-based contexts underpin the many ways in which the experiences and practices of managing a GI infection differed across areas and are explored below.

### 4.2 The extensive work required to clean up after a GI infection

The most common symptoms of GI infections - vomiting and/or diarrhoea - often created a huge amount of extra cleaning for families in both areas who participated in the study. Despite parents' best efforts, the mess and smell caused by these symptoms often spread over a wide area. Children, carpets, cushions, window sills, slats between bunk beds, parents' and children's beds, sheets, clothes, blankets, car seats and towels were all described as needing to be cleaned and washed, sometimes multiple times. This cleaning was particularly challenging for participants with younger, non-verbal, children who wore nappies which made managing diarrhoea more difficult and, as Annabelle, who lived in Seaview explained, ‘*can't tell you [they are] about to be sick*’ (Annabelle, Seaview). She and another participant living in Rockport who had a child who was two years old gave vivid descriptions of managing these symptoms:

*Yes, because it wasn't just being sick, it was literally shooting out his mouth I have never seen nothing like it in my life (…) it was like something off Scary Movie, it shot out his mouth. His nappy was like water (…) I had to put his swimming shorts [on], this sounds dead mad (…) otherwise he would leak outside of his nappy, so you know because swimming shorts are tight, these are new ones that stuck to him and it helped [it to stay in] the nappy because it was literally like dishwater it was that bad.* (Naomi, Rockport)

*[my son] would just stand there and be sick over your bed, your sofa, you, carpets … yes it was tough.* (Annabelle, Seaview)

As children often had symptoms which carried on into the night, parents became *‘exhausted’*, as they lost sleep while cleaning up the mess and comforting distressed children. Participants in both Rockport and Seaview described working hard to try to contain these dramatic, often physically violent symptoms using a variety of strategies to limit the mess. These strategies included: the use of swimming shorts to contain diarrhoea; putting children in the bath while they vomited; positioning containers such as buckets as close as possible to children; putting children who were just potty trained (and therefore no longer wearing nappies) back into nappies to contain diarrhoea; continually changing nappies; dressing children only in nappies to reduce washing; and putting children to sleep on towels to prevent bed-sheets becoming soiled.

The length and frequency of illness, as well as the age of the child, had a large impact on the work needed to manage illness as both increased the extent of cleaning. Many households, in both Rockport and Seaview had managed more than one of these infections in the previous 12 months:

*The kids pick them up from school quite often, (…) [my older daughter] doesn't go three months without one.* (Harriett, Seaview)

Two participants in Rockport had experienced a stomach bug at least once a month or every other month over the past year. Having these infections so close together meant that there was barely time to recover before the next bug arrived. One participant described how she coped with the impact of multiple GI infections:

*Don't really – should see my washing basket out there it is still like piled up from like last week. Two weeks ago. She is just constantly sick on everything bed sheets and things like that. Nightmare. (…), you find weeks after, you are still trying to catch up on washing and things and you are bloody knackered. It really drains me.* (Georgia, Rockport)

The amount of work required to clean up after the symptoms of a GI infection in young children was often, therefore, intensive and exhausting for parents in both settings. This work was not, however, the only kind of labour associated with having a GI infection. Below we describe other types of work involved in managing a GI infection and how inequalities in this work came about.

### 4.3 Inequalities in the work done while trying to prevent illness spreading

As parents described the cleaning involved in managing GI infections, it became clear that this cleaning was not limited to removing the mess and smell from the home. Cleaning was also used as a strategy to prevent other people from becoming ill. Vomit and diarrhoea were understood by participants to be infectious material which could make other people ill, a message reinforced by UK public health messaging about cleaning in the context of GI infections (NHS, 2020a). Participants in Rockport and Seaview described multiple strategies to try and get rid of the ‘bugs’ spread around the home which went above and beyond cleaning the mess. They described labour-intensive practices such as washing clothes multiple times, putting toys through the dishwasher, wiping down surfaces that ill children touched and washing furnishings and carpets using carpet cleaners. Importantly, this cleaning was made easier when there was access to certain resources. For example, having access to carpet cleaners and dishwashers, more evident in Seaview, enabled these participants to use less labour-intensive cleaning methods that were not available to all residents in Rockport:

*… we have an amazing dishwasher [which] kills pretty much everything [referring to GI bugs] I think.* (Jack, Seaview)

One practice that some participants used to prevent illness spreading was to physically separate members of the family who were ill from those who were not ill. This physical separation was much easier for Seaview's residents who often lived in much larger houses with multiple rooms both up and downstairs. Participants in Seaview described how they would put children who were ill in different rooms to those who were well, use separate bathrooms and put children to sleep in different bedrooms. In contrast, in Rockport, houses were smaller, often limited to one living room and kitchen downstairs and one bathroom and two bedrooms upstairs. There was not, therefore, the physical space to separate members of the family and everyone had to share bedrooms, living space and bathrooms. For families with one bathroom, if multiple people were ill at the same time, sinks and baths also had to be used to contain the vomit and diarrhoea. These physical contrasts are clear in the following descriptions from two parents, Linzi in Seaview and Freya in Rockport:

*They [her twins] slept in separate rooms when they were being sick, mainly just because they were disturbing each other, so I guess that probably might have helped to stop the spread of germs …* (Linzi, Seaview)

*I will get it the same time as her dad (…) And it is not nice when one is [vomiting] in the sink and one is in the toilet, really not nice.* (Freya, Rockport)

Households in Rockport who experienced frequent GI infections and whose smaller homes made it more difficult to separate family members went to extraordinary lengths to try and stop these incredibly disruptive infections. Lydia, who lived in Rockport, said that she bleached and disinfected the house *‘constantly’* because it was *‘too hard’* when she got sick as well as the children. Georgia said that she was so anxious to keep dirt away from her daughter to stop her getting another stomach bug that she now cleaned and mopped her floors three to four times a day, a laborious task she found exhausting:

*Yeh it is, it is really stressful. I am knackered. (…) having to clean up - mop and stuff three to four times a day …* (Georgia, Rockport)

Inequalities in the work to prevent illness spreading were therefore evident between the two areas. Rockport residents who experienced frequent infections had more cleaning to do by the nature of having these infections more frequently, created more cleaning while trying to keep these infections out of their home and often managed this labour without access to time-saving resources such as carpet cleaners or having the means to physically isolate family members who were ill from those who were well.

### 4.4 Inequalities in caring practices used to manage GI infections

While the labour expended to clean and prevent infections spreading took a huge amount of time and effort, it was not this that created the most anxiety for parents. Instead, it was the thought of children becoming more ill, which, for most parents was linked to the risk of dehydration (which can progress to shock and death if not treated (Elliott, 2007)) or to an exacerbation of pre-existing medical conditions. As a far higher percentage of Rockport residents had long-term medical conditions compared to Seaview (ONS, 2016) this created inequalities in the caring practices and anxiety associated with managing GI infections.

Parents often described how symptoms of vomiting and diarrhoea were made worse by eating and drinking and when children had a GI infection they often ‘*couldn't keep any water down …. ’* (Harriett, Seaview). Parents therefore worked hard to give their children fluids to prevent them becoming dehydrated using techniques which didn't immediately bring on vomiting. Many participants had learnt from past experiences with GI infections or from medical advice that the best way to do this was to give children very small amounts of fluids, often. Naomi explained how she followed this advice in practice by using small plastic dosing syringe from a child's ‘Calpol’ (liquid paracetamol) medication:

*… even if he had a little sip it will come straight back up, I had to get the Calpol syringes, I actually put juice in that and just give it him dead [really] slow because I wanted to make sure he had something and then I just had to literally put it on his tongue and just dead slow and then that way it stayed down. (…) And I just had to give him drinks like that the whole day …* (Naomi, Rockport)

Caring for a child or family member became even more difficult if the symptoms of a GI infection had to be managed alongside pre-existing health conditions. These health conditions often made either the GI infection, or the existing health condition worse. Pre-existing medical conditions such as diabetes, stomach ulcers, epilepsy, asthma, eczema, heart conditions, bowel conditions and clinical anxiety were all either thought to have exacerbated the symptoms of GI infections or to have made pre-existing health conditions more difficult to manage. Zoe, who lived in Rockport, described how her partner's diabetes symptoms had been exacerbated when he wasn't able to keep any food down to re-stabilise his blood sugars because he was being sick:

*[Partner] is type 2 diabetic so it can have quite significant effects on him where his blood sugars drop so low he's just completely groggy and out of it so I was trying to deal with him as well. He was more difficult to deal with than the baby [laughs].* (Zoe, Rockport)

These examples of symptoms being exacerbated by pre-existing medical conditions were overwhelmingly from residents of Rockport, reflecting the higher proportion of residents with a long-term health condition compared to Seaview (ONS, 2016). These more severe symptoms as a result of having a GI infection then created extra caring work and anxiety associated with managing GI infections for participants in Rockport compared to Seaview as they managed the labour-intensive symptoms of GI infections alongside these pre-existing health conditions.

### 4.5 Inequalities in the financial impact of illness

UK childcare policies provide parents in receipt of tax credits or state benefits with a free nursery place when their child turns two and all other children receive government funded nursery places when their child turns three (gov.uk, 2019b, 2019c). These criteria meant that the majority of participants in Rockport had access to a free nursery place whereas most participants in Seaview had at least one child who did not qualify for free childcare. UK public health policies, which require children to stay away from these nurseries and schools, and adults to stay away from work until at least 48 hours after symptoms of a GI infection stop, shaped inequalities in the financial consequences of illness between Rockport and Seaview. Even participants whose children were only ill for 24 hours were still expected to keep their child at home for an additional 48 hours after GI infection symptoms had stopped (NHS, 2020a):

*…. most of the time she is only sick, maybe twice, maybe three times, one night and by the next morning she is totally fine and eating food again and she has got to have the 48 hours off school but if it wasn't for that rule I would say she was ok to go in.* (Harriett, Seaview)

For others the illness lasted much longer, with one parent living in Rockport describing symptoms lasting 10 days and her daughter missing two weeks of nursery:

*[The GI infection] lasted for about 10 days, and then on that day, [referring to the day she met the researcher] because she'd had 48 hours without vomiting or anything we finally sent her back into school [nursery].* (Georgia, Rockport)

This emphasis on social isolation to prevent infection spreading therefore extended the length of time that these infections had an impact on families in the study. Keeping children at home in line with public health policies created inequalities in the financial consequences of illness which were shaped by contrasting financial and employment circumstances in the two areas. Participants and their partners in Seaview were generally employed in secure, salaried jobs that were reasonably well paid such as managerial posts, academia and medicine. These well-paid and secure jobs included arrangements for sick pay, carers pay and flexible working, all of which minimised the financial impact of GI infections even if participants did have to take time off work. Households in Seaview did not, therefore, describe financial hardship as a consequence of GI infections. Financial consequences were also minimised by having flexible working arrangements. Many households in Seaview had at least one parent who could arrange to work from home while also caring for their child:

*He [partner] is a civil servant. (…) he works flexi time so and they are much better, not that my work is not good, but his work is really good at letting him have days off to look after the kids. And he can also do a bit of work from home if he needs to so that is what he did.* (Penny, Seaview)

In contrast, participants and their partners in Rockport were often employed in less secure, less well-paid roles which were paid by the hour rather than salaried such as shop assistants, care assistants, cleaners or drivers. These jobs often did not allow flexible working which made taking time off work more difficult to arrange and often did not offer paid carer's leave or sick pay at a rate that compensated for a loss of pay. Many households in Rockport described loss of income or delays in pay as a consequence of time off work while having a GI infection as being challenging to manage. One household described the rate of statutory sick pay, (set at £94.25 per week at the time of fieldwork (gov.uk, 2019d)) as being half what they would usually get which made budgeting harder:

*… because my partner is the only one that works (…) if he has to take time off work, he only gets statutory sick pay so it is half of what he gets usually. Less than half, so it really affects us the following month because he has had to take time off work, he loses that next month.* (Lydia, Rockport)

Another participant who lived in Rockport worked as a carer and could not arrange at short notice for someone else to care for her client. Her inflexible employment, alongside her low income led her to decide that she had no option but to go to work after being up all night caring for her vomiting daughter, leaving her diabetic partner to look after their daughter at home:

*Oh, it was really, really difficult because I was so tired in work the next day, and we had to go to church in the morning and I'm sitting in church nearly falling asleep. But then at the same time still worrying about [daughter], thinking I've got to do this because I need the wages to pay my rent.* (Zoe, Rockport)

Living on a low income in Rockport also made the out-of-pocket expenses of having a GI infection more difficult to manage. Expenses such as paying for extra nappies, medication and buying new clothes to replace those that were soiled were challenging for participants living on a low household income in Rockport and were not mentioned by participants in Seaview. Talia and Scott, who lived in Rockport, explained that they kept money to one side so that if one of them was ever ill they could still pay travel costs to get to healthcare quickly, despite having access to free NHS healthcare:

*I think the good thing with us as well we always make sure there is money there, you know, for taxi fares in case we need to go up. So we always keep money to one side, even if we are not ill just in case something happens so we can get there quick.* (Scott, Rockport)

The financial consequences of having a GI infection could therefore be seen to contrast starkly across Rockport and Seaview. These consequences sat alongside inequalities in the labour expended to prevent other people becoming ill and disparities in pre-existing health conditions which shaped differences in the severity of symptoms and care.

## 5 Discussion

This ethnographic study has examined the practices surrounding, and lived experience of, GI infections which are often ‘hidden’ in the home in the UK. Our data demonstrate that although these infections were a disruptive event for most households, their consequences often had more wide-ranging and serious consequences for participants living in Rockport, a ‘disadvantaged’ area, than those living in the relatively ‘advantaged’ Seaview. These inequalities in the management and consequences of GI infections were underpinned by their wider social, economic, and environmental contexts. These inequalities were not, therefore, simply a consequence of individual choice and adherence (or non-adherence) to individual behavioural public health messages.

In demonstrating inequalities in the management and consequences of having a GI infection, we showed how participants in Rockport had to expend more effort to prevent infection spreading, in caring for children and experienced more financial consequences as a result of having a GI infection in the household. Our study shows how the ‘work’ needed to manage gastrointestinal infections is more laborious for people living in more ‘disadvantaged’ conditions, exacerbated by: more overcrowded homes with fewer washing and toilet facilities; inflexible employment; low household incomes; and higher likelihood of co-morbidities which can be made worse by having a gastrointestinal infection. Our findings call into question the dominant approach to GI infections which focuses on individual behaviours to reduce risk (Rotheram et al., 2020). This study has also shown how the huge amount of work required to manage a GI infection sits in contrast to current dominant descriptions of many GI infections as ‘acute’, ‘self-limiting’ diseases which suggests an illness of short duration which is easily managed in the home (Tam et al., 2012). Instead, our findings show how the lived experience of acute GI infections has much in common with sociological literature describing the labour needed to manage a chronic illness (e.g. Corbin and Strauss, 1985; Mattingly et al., 2011; Burton, 2000). The practice of giving small amounts of water to prevent dehydration in particular, displays the kind of expert knowledge previously linked to skills needed to care for people with chronic conditions rather than managing shorter illnesses (Corbin and Strauss, 1985; Mattingly et al., 2011).

Descriptions of physically cleaning up vomit and diarrhoea have much in common with the ‘dirty work’ described in sociological research on the cleaning of urine and faeces done by nursing staff in hospitals and nursing homes (Jervis, 2004). In effect, families with GI infections can be understood to become informal healthcare providers within their own home (Cooper et al., 2016) and develop expertise, over time, to manage these infections. We argue, therefore, that descriptions of acute, self-limiting illnesses, understate the impact that GI infections have for families with young children and in the process hides inequalities in the consequences of illness.

### 5.1 Strengths and limitations

As with any study, there were limitations to the collected data. Firstly, it was necessary to carry out interviews while young children were present. This created challenges in interviewing and may have contributed to the relative brevity of interviews (30 min or less). However, by using narrative pointed questions and combining interviews with other data collected through observations it was still possible to access the lived experience of managing GI infections and, importantly, allowed families with young children to take part. Secondly, in using a place-based approach with specific boundaries, this research may have missed data which could have been collected had a more traditional ethnographic approach without ward boundaries been used (Hammersley and Atkinson, 2007). The use of boundaries, did, however allow for comparisons to be made between contrasting areas, to address our central research question of how socioeconomic inequalities in the management and consequences of GI infections come about.

Another important limitation is the dominance of female voices in this study. While we did not draw out the gendered nature of the labour in managing GI infections, the prevalence of female voices fits with other research which has found women bear the worst emotional and physical consequences of GI infections (McGarrol et al., 2020) as well as emerging COVID-19 research which finds women carry the brunt of childcare responsibilities during lockdowns (Wenham et al., 2020). The gendered nature of care in infectious diseases would be an important area of future research.

One ethical dilemma we reflected on was the importance of our research into GI infections (often associated with dirt and lack of good hygiene (Meah, 2014)) avoiding adding to the stigma which ‘disadvantaged’ communities already experience through living in places described as ‘deprived’ (Garthwaite and Bambra, 2018). An important strength of this study was that by focusing on the wider social, economic and place-based context of inequalities in GI infections our insights may avoid adding to this stigma or associating differences in GI infections between socioeconomically contrasting places with notions of poor hygiene practices. These strengths suggest ethnography would be a useful approach for future studies wishing to examine inequalities in other infectious diseases such as COVID-19.

### 5.2 Policy implications

Our findings point to the importance of UK policies for GI infections moving away from solely considering individual behaviours around risk, to also consider the wider context of the management and consequences of GI infections. Public health agencies need to consider how wider social, economic and policy contexts can shape inequalities in acute infectious diseases if they wish to address (and avoid exacerbating) inequalities in the future. The study also provides further evidence of the value of lay knowledge and what we can learn from the experiences and practices of lay people.

Importantly, our findings, while specific to GI infections, may also be pertinent to other acute infectious diseases such as COVID-19, for which public health responses have also focused on reducing the spread of infection through individual behaviours such as hand hygiene and social isolation (NHS, 2020b). While these public health approaches are clearly vital to reduce the spread of infectious diseases, we have shown, using the example of GI infections, that they are not the only important consideration for those concerned with preventing or ameliorating inequalities. Our findings demonstrate how pre-existing health conditions, disproportionately found in ‘disadvantaged’ communities can act synergistically with infectious diseases to worsen the symptoms of illness. We show how differences around household resources, housing, working conditions and income create inequalities in the consequences of infectious diseases beyond those of clinical symptoms. Socioeconomic inequalities in the consequences of COVID-19 infections are already emerging in the UK (ONS, 2020) and an argument is being put forward for locally adaptive responses to be developed in partnership with communities (Rose et al., 2020). Our research supports a move towards interventions which reflect the lived experience and consequences of managing illness in different socioeconomic circumstances rather than ‘one size fits all’ national interventions focusing solely on reducing risk. Research, legislation and interventions must be aware of, and address, structural causes of the consequences of infectious diseases to prevent a further widening of inequalities and ‘blaming’ of communities as COVID- 19 spreads around the globe.

## Credit author statement

Suzanne Rotheram: Conceptualisation, Methodology, Formal analysis, Investigation, Writing – original draft preparation. Jessie Cooper: Conceptualisation, Methodology, Formal analysis, Writing – review & editing, Supervision. Ben Barr: Conceptualisation, Methodology, Formal analysis, Writing – review & editing, Supervision. Margaret Whitehead: Conceptualisation, Methodology, Formal analysis, Writing – review & editing, Supervision, Funding acquisition.
